# Maternal knowledge explains screen time differences 2 and 3.5 years post-intervention in INFANT

**DOI:** 10.1007/s00431-021-04134-8

**Published:** 2021-06-01

**Authors:** Christine Delisle Nyström, Gavin Abbott, Adrian J. Cameron, Karen J. Campbell, Marie Löf, Jo Salmon, Kylie D. Hesketh

**Affiliations:** 1 Department of Biosciences and Nutrition, Karolinska Institutet, Neo, 141 83 Huddinge, Sweden; 2 Institute for Physical Activity and Nutrition (IPAN), Deakin University, Geelong, 3125 Australia; 3 Global Obesity Centre, Institute for Health Transformation, Deakin University, Geelong, 3125 Australia; 4 Department of Health, Medicine and Caring Sciences, Division of Society and Health, Linköping University, 581 53 Linköping, Sweden

**Keywords:** Long-term follow-up, Knowledge, Paediatrics, Screen time

## Abstract

Very few early childhood interventions have observed sustained effects regarding television viewing and none have examined the mechanisms behind sustained intervention effects at long-term follow-ups. Thus, the aim of this study was to investigate potential mechanisms relating to the maintained intervention effect on television viewing at two long-term follow-ups in the Melbourne Infant Feeding Activity and Nutrition Trial (INFANT). INFANT was a cluster-randomised controlled trial. At the 2- and 3.5-year follow-ups, a total of 262 infant/mother pairs had complete information. Television viewing was assessed via a questionnaire at both follow-ups and six potential mediators were measured post-intervention (i.e. 15 months after baseline). Causal mediation analysis was conducted. At the 2- and 3.5-year follow-ups, the positive impacts of INFANT on maternal television viewing knowledge were maintained (B = 0.34 units; 95% confidence interval (CI_95_): 0.21, 0.48). An indirect effect of the intervention on reducing children’s television viewing time was observed at the 2- and 3.5-year follow-ups (B = −11.73 min/day; CI_95_: −22.26, −3.28 and B = −4.78 min/day; CI_95_: −9.48, −0.99, respectively) via improved maternal television viewing knowledge.

*Conclusion*: The positive impacts of INFANT on maternal television viewing knowledge were maintained at both follow-ups, with better maternal knowledge associated with less television viewing time in their children. These results have implications for paediatricians and healthcare professionals as educating new parents early on regarding screen time may lead to the development of healthier screen time habits that are sustained through to the pre-school years.

**Table:** 

**What is Known:** • *Lifestyle behaviours inclusive of screen time have been found to be established before the pre-school years and track.* • *Few trials have evaluated the long-term mechanisms related to maintained intervention effectiveness.*	
**What is New:** • *This study shows the positive impacts of a low-dose intervention on maternal television viewing knowledge at two long-term follow-ups.* • *Better maternal television viewing knowledge was associated with less television viewing time in their children.*

**What is Known:**

• *Lifestyle behaviours inclusive of screen time have been found to be established before the pre-school years and track.*

• *Few trials have evaluated the long-term mechanisms related to maintained intervention effectiveness.*

**What is New:**

• *This study shows the positive impacts of a low-dose intervention on maternal television viewing knowledge at two long-term follow-ups.*

• *Better maternal television viewing knowledge was associated with less television viewing time in their children.*

## Introduction

There are ongoing debates between clinicians, researchers, policy makers, and parents regarding the benefits versus risks of children’s screen time exposures [1]. Screens (e.g. television, tablets, and smartphones) are ubiquitous in today’s society and pre-school-aged children are well exceeding the recommendation of 1 h or less per day [2–4]. A recent systematic review and meta-analysis found strong evidence for associations between high amounts of screen time with overweight and obesity and shorter sleep duration in toddlers and pre-schoolers [5]. Furthermore, with regards to sleep duration and screen time, another systematic review and meta-analysis has also found unfavourable associations in infants and toddlers [6]. Excessive screen time has also been found to be associated with poor dietary outcomes, worse motor and executive function development, more aggressive behaviours as well as worse emotional and behavioural outcomes in children aged 0 to 7 years [5]. Since lifestyle behaviours, which include screen time, have been shown to be established within the first 2 years of life and track into the pre-school years [7–10], there is a need to promote healthy screen time habits early in life.

Interventions designed to promote healthy lifestyle behaviours in the early years are more likely to be effective if they involve parents and aim to improve their knowledge, skills, and competencies [11]. Furthermore, two cross-sectional studies have found that greater knowledge regarding screen time guidelines has been shown to be associated with lower television viewing in young children [12, 13]. Thus, understanding opportunities to influence screen time is important.

The Melbourne Infant Feeding Activity and Nutrition Trial (INFANT) was a community-based obesity prevention intervention targeting first-time Australian parents with a 4-month-old infant [14, 15]. With regards to screen time post-intervention (i.e. 15 months after baseline), an estimated 16 min/day reduction in television viewing time was observed [16]. Furthermore, while the confidence intervals spanned zero, there was some evidence of an effect at both long-term follow-ups with estimated reductions in television viewing time of approximately 10 min/day [17]. To date, very few early childhood interventions have observed sustained effects on television viewing and none have examined the mechanisms behind sustained intervention effects at long-term follow-ups. Thus, the aim of this study was to investigate potential mechanisms relating to the maintained intervention effect on television viewing at the 2- and 3.5-year follow-ups in INFANT.

## Methods

### Study design and participants

INFANT was a two-arm, community-based cluster-randomised controlled trial which took place in Victoria, Australia between 2008 and 2010. Details regarding the trial have been described previously [14, 16]. In brief, the intervention arm received INFANT (six group sessions plus take-home material over 15 months) in addition to usual care from maternal and child health services; the control group received usual care from maternal and child health services. With regards to screen time, INFANT targeted parental knowledge through the provision of information about screen time recommendations as well as discussion with participating parents that there are no proven benefits of screen time for infants and its potential detriments to aspects of development. In addition, alternative ideas for non-screen-based activities (e.g. active play ideas appropriate for the age of the child) as well as strategies for limiting screen time were provided [14]. Approval to conduct this study was received from the Deakin University Human Research Ethics Committee (2007-175).

In total, 542 children were randomised into INFANT, with 492 completing post-intervention measurements (91%) when the infants were on average 20 months of age. All families who completed the trial post-intervention were asked to participate in the long-term follow-ups 2 and 3.5 years post-intervention, when the children were on average 3.6 and 5 years old [15].

### Measures

This study utilised data from baseline, post-intervention, and the 2- and 3.5-year follow-ups. All data was collected using questionnaires filled out by the mothers and was collected in the same way at each time point.

#### Demographic information

When the families were first enrolled in the study, mothers filled in a questionnaire regarding demographic information for themselves and their infant.

#### Television viewing

At baseline as well as at the 2- and 3.5-year follow-ups, the amount of time spent watching television was assessed using the following two questions: ‘on an average weekday (Monday–Friday) how much time does your child spend watching or in front of the television?’ and ‘on an average weekend day (Saturday–Sunday) how much time does your child spend watching or in front of the television?’. The mothers then filled in the number of hours and minutes their child spent watching television on week and weekend days. In a separate sample of 47 Australian parents with a child aged 3 to 5 years, the questions have been found to have acceptable reliability (intraclass correlation coefficient 0.69, CI_95_: 0.54, 0.80) [18].

#### Potential mediators

At baseline and post-intervention, mothers filled in a questionnaire regarding potential mediators of television viewing. A total of six potential mediators of television viewing were measured, with five summary scores derived from a total of 32 separate questions. These were (i) self-efficacy regarding television viewing (i.e. parental confidence to limit children’s television viewing [3 questions]); (ii) facilitating television viewing (i.e. over the past month the frequency of parents facilitating their child’s television viewing, for instance turning on the television [7 questions]); (iii) television viewing knowledge (i.e. agreement with statements regarding detriments and recommendations relating to children’s television viewing [4 questions]); (iv) use of television (i.e. agreement with statements on how parents use television in relation to their child [5 questions], e.g. ‘I use TV to distract my child when s/he is being difficult’, ‘I use TV to keep my child occupied so that I can get things done’); and (v) sedentary behaviour in the home environment (i.e. indication of whether the child has access to items/environments that promote screen use [13 questions]). The sixth mediator was future expectations of parents relating to their child’s television viewing habits when they are older and it was derived from two questions. In a separate sample of parents, test-retest reliability showed acceptable agreement [19] for most items (85% weighted kappa > 0.4 at baseline; 75% weighted kappa > 0.4 post-intervention).

#### Statistical analyses

**Fig. 1:**
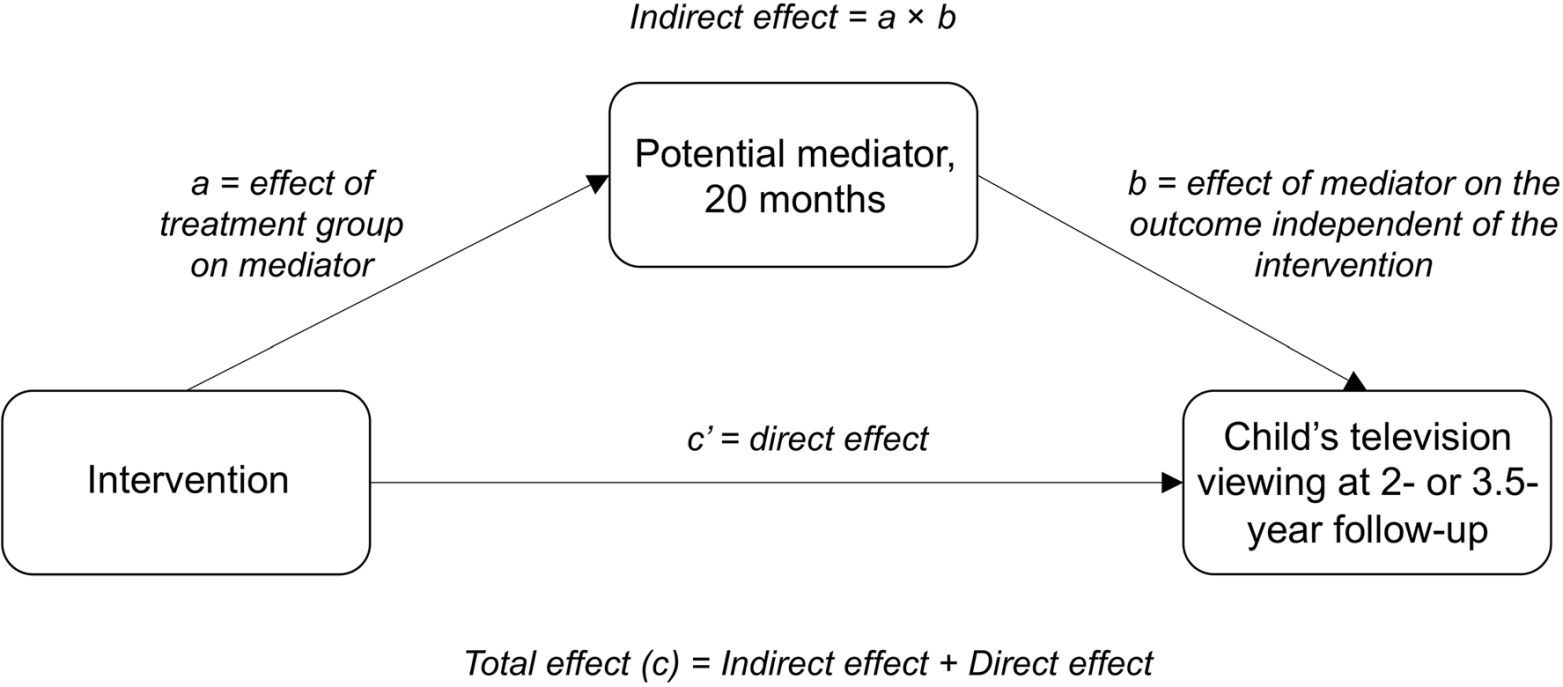
Illustration of the hypothesised mediating pathways

Mother and child dyads were only included in the analyses if they had complete data for all of the aforementioned measures. Descriptive statistics were used to present the baseline characteristics of the participating children and mothers at the 2- and 3.5-year follow-ups. Causal mediation analysis was conducted for potential mediators for which there was a statistically significant intervention effect at 20 months [20, 21]. Figure 1 illustrates the hypothesised mediating pathways. Intervention effects on potential mediators were assessed by fitting linear regression models for the potential mediators when the infants were 20 months old (i.e. post-intervention), while adjusting for child sex, maternal education, and the baseline mediator value (*a* path). Separate linear regression models were fitted to test the effect of each potential mediator when the infants were 20 months old, on television viewing time at 3.6 and 5 years of age, while adjusting for treatment group, child sex, maternal education, and the baseline mediator value (*b* path). Following the causal inference approach to mediation [20, 21], *b* path models initially included an interaction term between the treatment group and the potential mediator, to allow that the effects of the mediator on the outcome may differ across treatment groups. When the p-value for the interaction term was ≥ 0.05, the interaction term was omitted. Causal mediation models with selected potential mediators were run using the Stata package *medeff* [22] using 5000 simulations. All models were fitted with cluster-robust standard errors to account for clustering of participants in the parent groups. All coefficients presented are unstandardized. Stata (Release 15, StataCorp LP, College Station, TX, USA) was used to conduct all analyses and statistical significance was set at p < 0.05.

## Results

Table 1 presents the baseline characteristics of the participating children and mothers at the 2- and 3.5-year follow-ups. A total of 262 infant/mother pairs had complete information at both the 2- and 3.5-year follow-ups. Table 2 presents the results of models assessing intervention effects on potential mediators (*a* path). There was strong evidence of an intervention effect on the intervention group’s television viewing knowledge when compared to the control group (B = 0.34 units; CI_95_: 0.21, 0.48), but limited evidence of intervention effects on the remaining potential mediators.

**Table 1:** Demographic characteristics of the mothers and infants at baseline (n = 262)^a^

	Mean ± SD or (n, %)
Infants	
Age (months)	3.6 ± 1.0
Male	141 (54%)
Mothers	
Age (years)	32.6 ± 4.2
Education level	
Low (completed up to final year of 2° school)	47 (18%)
Middle (completed trade/certificate postsecondary school)	55 (21%)
High (completed university degree or beyond)	160 (61%)

*SD*, standard deviation

^a^Includes all participants who had complete data at both the 2- and 3.5-year follow-ups

**Table 2:** Estimated effect of the intervention on potential mediators (*a* path) (n = 262)

Potential mediators^a^	B (CI_95_)^b^	p-value
Self-efficacy around television viewing	0.10 (−0.07, 0.27)	0.256
Facilitating television viewing	−1.01 (−4.54, 2.53)	0.570
Television viewing knowledge	0.34 (0.21, 0.48)	<0.001
Use of television	−0.09 (−0.23, 0.04)	0.176
Sedentary behaviour in the home environment	0.14 (−0.18, 0.45)	0.387
Future expectancies	−0.10 (−0.23, 0.03)	0.114

Abbreviations: *CI*_*95*_, 95% confidence interval

^a^Presented as summary scores

^b^Estimated mean difference between intervention and control group, and adjusted for child sex, maternal education, and the baseline mediator value

### 2-year follow-up

Associations between facilitating television viewing, television viewing knowledge, future expectancies, and sedentary behaviour in the home environment with children’s television viewing time (*b* path) were observed (Table 3). An interaction was found between facilitating television viewing and treatment group (p = 0.004) for children’s television viewing time. Facilitating television viewing was positively associated with increased television viewing time in the control group (B = 2.18 min/day; CI_95_: 1.06, 3.31), while no association was found in the intervention group (B = −0.53 min/day; CI_95_: −2.06, 1.00). As no other interactions were found by treatment group, the remaining results are not stratified. Lower television viewing time was observed in children of mothers with greater television viewing knowledge (B = −33.92 min/day; CI_95_: −57.51, −10.33). Additionally, lower television viewing time was observed for those whose mothers expected their child to have similar television viewing habits to their own (B = −34.01 min/day; CI_95_: −58.14, −9.88). Finally, higher television viewing time was found in children with more access to items or an environment that promotes screen time (B = 7.69 min/day, CI_95_: 0.01, 15.36).

**Table 3:** Estimated effects of potential mediators on children’s television viewing time (*b* path) at the 2- and 3.5-year follow-ups (n = 262)

Potential mediators^a^	2-year follow-up^c^		3.5-year follow-up	
	B (CI_95_)^b,c^	p-value	B (CI_95_)^b^	p-value
Self-efficacy around television viewing	0.90 (−18.16, 19.96)	0.925	−5.53 (−14.65, 3.59)	0.230
Facilitating television viewing	Intervention: −0.53 (−2.06, 1.00)	0.491	1.15 (0.62, 1.68)	<0.001
	Control: 2.18 (1.06, 3.31)	<0.001		
Television viewing knowledge	−33.92 (−57.51, −10.33)	0.006	−13.85 (−24.66, −3.05)	0.013
Use of television	−11.24 (−43.99, 21.52)	0.495	9.75 (−3.04, 22.55)	0.133
Sedentary behaviour in the home environment	7.69 (0.01, 15.36)	0.050	−0.68 (−5.92, 4.56)	0.797
Future expectancies	−34.01 (−58.14, −9.88)	0.007	−10.45 (−25.22, 4.31)	0.162

Abbreviations: *CI*_*95*_, 95% confidence interval

^a^Presented as summary scores

^b^Adjusted for child sex, maternal education, and the baseline mediator value

^c^Where there was evidence of an interaction between treatment group and the potential mediator at the p < 0.05 level, separate effects estimates are presented for the intervention and control groups

For the 2-year follow-up, only television viewing knowledge met the criteria (i.e. a statistically significant *a* path) to be included in the causal mediation analysis. No statistically significant direct effect (*c’* path) or total effect (*c* path) of the intervention on children’s television viewing was found (B = −4.06 min/day, CI_95_: −33.08, 25.52 and B = −15.80 min/day, CI_95_: −42.82, 11.06, respectively). However, a statistically significant indirect effect of the intervention on children’s television time viewing, via improved maternal television viewing knowledge, was observed (B = −11.73 min/day; CI_95_: −22.26, −3.28).

### 3.5-year follow-up

Associations were observed between facilitating television viewing and television viewing knowledge with children’s television viewing time (*b* path). Greater facilitation of television viewing by mothers was associated with higher television viewing time in the children (B = 1.15 min/day; CI_95_: 0.62, 1.68). Furthermore, greater maternal television viewing knowledge was associated with children watching less television (B = −13.85 min/day; CI_95_: −24.66, −3.05).

Only television viewing knowledge met the criteria to be included in the causal mediation analysis at the 3.5-year follow-up. No statistically significant direct (*c’* path) or total effect (*c* path) of the intervention on children’s television viewing was found (B = −7.00 min/day; CI_95_: −24.27, 10.61 and B = −11.78 min/day; CI_95_: −28.84, 5.52, respectively). However, a statistically significant indirect effect of the intervention on children’s television viewing time via improved maternal television viewing knowledge was observed (B = −4.78 min/day; CI_95_: −9.48, −0.99).

## Discussion

There is strong evidence that there were sustained positive effects of INFANT on maternal television viewing knowledge at both the 2- and 3.5-year follow-ups, with better maternal television viewing knowledge being associated with less television viewing time in their children at both time points. Despite the lack of maintained significant intervention effects on children’s television viewing over time, there is evidence of a statistically significant indirect effect of INFANT on reduced television viewing via improved maternal television viewing knowledge.

To date, no interventions in young children have investigated the potential mechanisms related to sustained intervention effects regarding television viewing. In school-aged children and adolescents, two systematic reviews investigated mediating mechanisms in obesity prevention interventions; however, too few studies have been conducted with regards to sedentary behaviour (including screen time) to draw clear conclusions [23, 24]. With regards to behaviour change, knowledge alone is usually not enough to evoke sustained change [25]; however, it is likely that a lack of knowledge may limit or prevent behaviour change from occurring. As the first years of a child’s life have been found to be when parents are most receptive to information regarding how to best guide their child [26], this could be the time to target parents and provide them with important information regarding screen time usage. In the current study, parents in the intervention group received information regarding television viewing when their child was approximately 4–19 months of age. This low-dose intervention was enough to increase maternal television viewing knowledge and subsequently reduce children’s television viewing time with the effect maintained at both long-term follow-ups. Thus, as maternal television viewing knowledge remained higher in the intervention group compared to the control group, the sustained impact on knowledge is likely what led to the reduction in television viewing at both follow-ups. Furthermore, as INFANT was based on the anticipatory guidance approach [14], it could also be hypothesised that the indirect intervention effect on television viewing via improved maternal television viewing knowledge at both follow-ups could be due to the knowledge gained during the intervention provided the parents with the tools to handle situations regarding television viewing when their child was older.

Interestingly, at the 2-year follow-up, we found that the intervention had a moderating effect on facilitating television viewing (e.g. turning the television on). In the control group, facilitating television viewing was positively associated with television viewing time, whereas no association was observed in the intervention group. Even though the intervention had no discernible impact on facilitating television viewing (*a* path), it appears that the intervention has reduced the impact of facilitating television viewing on television viewing time. The lack of an association between facilitating television viewing and television viewing time observed in the intervention group may be due to the mothers’ increased knowledge of screen time recommendations. In a cross-sectional study by Goh et al. [13], increased parental knowledge regarding screen time recommendations was associated with lower levels of screen viewing in infants. Furthermore, in European pre-school-aged children parents, being knowledgeable regarding screen time guidelines was associated with low television viewing [12]. Thus, in the current study, it can be hypothesised that the increased maternal knowledge from INFANT led mothers to more effectively limit television viewing.

This study employed a strong cluster-randomised controlled trial design with assessment of intervention effects at two additional follow-ups. A further strength of this study is the broad range of potential mediators assessed. Furthermore, the use of a score for each potential mediator rather than an individual question is another strength. The use of parent-reported television time is a potential limitation; however, the questions used have been found to have acceptable reliability [18]. Furthermore, when the study was initiated in 2008, other types of screen time (e.g. tablets and smartphones) were not as prevalent as they are today and thus this study focused on television viewing. Finally, the small sample size, the fact that the sample had a higher proportion of mothers with greater educational attainment, as well as the inclusion of only first-time parents limits the generalizability of the findings.

This study has implications for paediatricians and other healthcare professionals, as it provides evidence that educating new parents early on regarding screen time recommendations and potential detriments may reduce screen time in the pre-school years. A recent study by Belay et al. [27] found that compared to 2006, in 2017 significantly more paediatricians talked to parents regarding screen time. These are promising findings; however, these conversations occurred when the children were over 2 years of age [27]. With the digitalization of today’s world, screens are ubiquitous throughout all age groups. Thus, there is a need for paediatricians and other healthcare professionals to educate parents even earlier regarding healthy screen time habits.

## Conclusion

The positive effects of INFANT on maternal television viewing knowledge were maintained at both the 2- and 3.5-year follow-ups, with better maternal television viewing knowledge being associated with less television viewing time in their children. Despite the lack of a statistically significant sustained total intervention effect on television viewing, there is evidence of an indirect effect of INFANT on reduced television viewing via improved maternal television viewing knowledge. These results have implications for paediatricians and other healthcare professionals, as educating new parents early on regarding screen time may lead to the development of healthier screen time habits in infancy and the pre-school years.

## Data Availability

De-identified individual participant data will not be made available.

## Abbreviations

INFANT: Melbourne Infant Feeding Activity and Nutrition Trial
CI_95_: 95% confidence interval
